# Assessing psychometric challenges and fatigue during the COVID-19 pandemic

**DOI:** 10.25122/jml-2023-0244

**Published:** 2023-10

**Authors:** Arstan Mamyrbaev, Anar Turmukhambetova, Saule Bermagambetova, Umit Satybaldieva, Gulmira Erimbetova, Kulyan Shayakhmetova, Gulsim Karashova, Marat Iztleuov, Ibrahim Abdelazim

**Affiliations:** 1 Department of Hygienic Disciplines and Occupational Diseases, West Kazakhstan Marat Ospanov Medical University, Aktobe, Kazakhstan; 2 Karaganda Medical University, Karaganda, Kazakhstan; 3 Department of Natural Sciences, West Kazakhstan Marat Ospanov Medical University, Aktobe, Kazakhstan; 4 Department of Obstetrics and Gynecology, Faculty of Medicine, Ain Shams University, Cairo, Egypt

**Keywords:** Covid-19, fatigue syndrome, pandemic, psychometric, Covid-19: Coronavirus Disease-19, GF: General fatigue, KMU: Karaganda Medical University, MF: Mental fatigue, MFI-20: Multidimensional Fatigue Inventory-20, OR: Odds ratio, PhF: Physical fatigue, RA: Reduced activity, RM: Reduced motivation, SARS: Severe acute respiratory syndrome, WHO: World Health Organization, χ^2^: Chi-square test

## Abstract

Environmental hazards and/or pandemics may push humans to use different protective methods to maintain their well-being. This study aimed to identify populations vulnerable to psychometric challenges and fatigue during the Coronavirus Disease (COVID-19) pandemic in Kazakhstan. A total of 1,592 participants were recruited and asked to complete the Multidimensional Fatigue Inventory-20 (MFI-20). Participants were classified according to gender and age. Data were analyzed using the Chi-square test (x2) and MedCalc to detect the odds of MFI-scales with a score ≥12 in women compared to men. Young women had significantly higher odds (OR) of reduced activity (OR 2.4, p<0.0001), physical (OR 2.5, p<0.0001), and mental fatigue (OR 3.4, p<0.0001) than young men. Middle-aged women had significantly higher odds of general fatigue (OR 2.1; p<0.0001), reduced motivation (OR 2.1, p<0.0001), physical (OR 2.1, p<0.0001), and mental fatigue (OR 1.9, p<0.0001) than did middle-aged men. Elderly women had significantly higher odds of general fatigue (OR 3.6, p<0.0001), reduced motivation (OR 3.5, p<0.0001), and physical fatigue (OR 3.5, p<0.0001) than men in the same age category. The study highlights that women were more susceptible, with significantly increased odds of experiencing various aspects of fatigue syndrome compared to men during the COVID-19 pandemic in Kazakhstan. Therefore, it is necessary to investigate individual behavioral changes to help identify vulnerable populations and provide relevant evidence for developing protocols and guidelines during pandemics and/or outbreaks.

## INTRODUCTION

In 2019, several cases of novel Coronavirus Disease-19 (Covid-19) pneumonia were reported in China [1]. By February 2020, as Covid-19 began spreading beyond China's borders, the World Health Organization (WHO) officially declared it a pandemic [2]. This global health crisis led to an increased number of fatalities, prompting governments to implement preventive measures to control the spread of Covid-19 [3]. In the Republic of Kazakhstan, the Presidential Decree (No. 285 in March 2020) introduced preventive measures to limit the spread of Covid-19 throughout the country.

Research on severe acute respiratory syndrome (SARS) outbreaks has highlighted the impact of such events on stress levels across various age groups, professional sectors, and geographic regions [4-7]. Environmental hazards and/or pandemics may push humans to use different protective methods to maintain their well-being and mitigate stress or hazards [3]. A study by Dorfan and Woody [8] reported several behavioral changes during the SARS outbreak, including avoidance, anxiety, the urge to wash, and increased wipe consumption. Studies regarding knowledge, attitudes, and preventive behaviors during the COVID-19 pandemic have reported an association between levels of knowledge and intention toward preventive measures [9-11]. Furthermore, studies on Covid-19 also reported substantial mental health issues (i.e., depression and anxiety) associated with the pandemic [12-16]. Some studies found that young individuals with higher education had a higher level of stress during the Covid-19 pandemic [17]. Chinese research found that access to relevant medical information during the COVID-19 pandemic reduced anxiety and stress [18, 19].

The Covid-19 pandemic is the most crucial event in the 21^st^ century. Fear of exposure and/or infection with Covid-19 resulted in unreasonable behavior in most countries. While previous studies have primarily focused on investigating preventive measures against COVID-19 in relation to participants' knowledge, there remains a notable gap in research concerning individual behavioral changes during the COVID-19 pandemic. Understanding how individual behavior changed during the COVID-19 pandemic is crucial for identifying vulnerable populations who may experience psychological distress and fatigue. In this cross-sectional study, we aimed to investigate these vulnerabilities during the pandemic to inform the development of more effective protocols and guidelines for pandemic and outbreak management.

## MATERIAL AND METHODS

A number of 1,592 participants aged between 18 and 75 years were recruited for this cross-sectional cohort study, conducted over two years (2021 and 2022) in Karaganda, Republic of Kazakhstan. The study aimed to identify populations vulnerable to psychometric challenges and fatigue during the COVID-19 pandemic. The study was conducted at the Karaganda State Medical University (KMU) hospital, the largest tertiary medical center in Karaganda, following the requirements of the Republic of Kazakhstan as a modern education medical center offering integrated academic health services. The KMU hospital contains out-patient clinics with structural subdivisions (i.e., consultation and diagnostic centers), day-care minor and major surgery units, postoperative hospital stay facilities, and 24-hour emergency services.

Inclusion criteria comprised individuals aged 18-75 years old who could communicate and had a self-reported history of COVID-19 infection. Exclusion criteria included individuals below the age of 18 or above 76 years, those with communication difficulties or the inability to self-report, and those who declined or were unable to provide informed consent.

Demographic information, including age, gender, and current work status of participants, were collected. Participants were asked to complete the Multidimensional Fatigue Inventory-20 (MFI-20) to evaluate psychometric challenges and fatigue during the Covid-19 pandemic. The MFI-20 is a 20-item self-reported instrument developed by Smets *et al*. [20] to measure fatigue and psychometric behavior. This instrument was used to quantitatively assess different aspects of fatigue and its severity [20]. The MFI-20 covers the following five scales: general fatigue (GF), physical fatigue (PhF), mental fatigue (MF), reduced motivation (RM), and reduced activity (RA). Each scale consisted of four items with responses rated on a 5-point Likert scale (1 = Disagree Strongly, 2 = Disagree, 3 = Neither Disagree nor Agree, 4 = Agree, and 5 = Agree Strongly). Fatigue syndrome was diagnosed at a score of ≥12 for any of the five scales of the MFI-20, and a higher score indicated a higher level of fatigue (severe), while a lower score indicated a lower level of fatigue. The MFI-20 was designed to prevent and minimize the influence of subjective factors on participants [20] and has demonstrated reliability in measuring fatigue in the general population [21].

Participants were classified by gender (men and women) and age (young: >18-44 years, middle-aged:>44-60 years old, and elderly: >60-75 years) [22]. The collected data were analyzed using Chi-square test (x2) and MedCalc 20.106 (MedCalc., Belgium) to identify populations vulnerable to psychometric challenges and fatigue during the Covid-19 pandemic in the Republic of Kazakhstan, with a significance level set at p<0.05. The G Power 3.1.9.7 (Düsseldorf; Germany) [23, 24] was used to calculate the sample size of this study, with 0.05 probability, 0.95% power, and 0.3 sample size. The Chi-square test (x2) was used for statistical analysis.

## RESULTS

### Participant characteristics

A total of 1,592 participants, aged between 18 and <76 years old, from Karaganda, Republic of Kazakhstan, were included in this cross-sectional cohort study to identify populations vulnerable to psychometric challenges and fatigue during the COVID-19 pandemic using the MFI-20. Table 1 presents the participants’ characteristics. The proportion of employed men (61.3%) was significantly higher than that of women (48.6%) (p=0.0057), whereas the percentage of retired women (24.7%) was significantly higher than that of men (15.3%) (p=0.0001). Moreover, the percentage of men with a history of previous COVID-19 infection (36.7%) was significantly higher than that of women with such a history (29.2%) (p=0.02).

**Table 1:** Participants characteristics

Variables	Women (n=801)	Men (n=791)	p-value (X^2^ test)
**Age**			
Young age (n=447)	27.84% (223/801)	28.3% (224/791)	p=0.87
Middle age (n=695)	43.45% (348/801)	43.9% (347/791)	p=1.0
Elderly (n=450)	28.71% (230/801)	27.8% (220/791)	p=0.76
**Current working status**			
Working (n=874)	48.6% (389/801)	61.3% (485/791)	p=0.0057*
Not working (n=289)	20.2% (162/801)	16.0% (127/791)	p=0.07
Retired (n=319)	24.7% (198/801)	15.3% (121/791)	p=0.0001*
Individual Entrepreneur (n=57)	3.5% (28/801)	3.7% (29/791)	p=0.86
Student (n=53)	2.99% (24/801)	3.7% (29/791)	p=0.4
**Previous Covid-19 infection (n=524)**	29.2% (234/801)	36.7% (290/791)	p=0.02*
**No previous Covid-19 infection (n=1068)**	70.8% (567/801)	63.3% (501/791)	p=0.16

Data is presented as numbers and percentages (%). Elderly: >60-75 years old. Middle-age: >44-60 years old. X^2^: Chi-square test used for statistical analysis. Young age: >18-44 years old.

### Assessment of psychometric challenges and fatigue in different age groups

**Figure 1:**
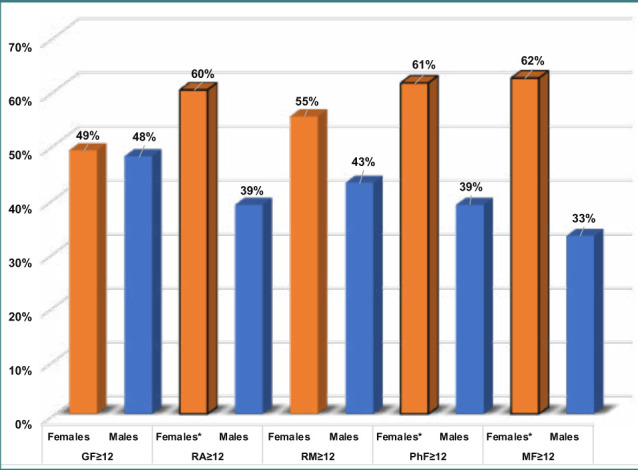
MFI-scales (score ≥12) among young women compared to men *: Significantly different percentages between women and men with scores ≥12 on the RA, PhF, and MF scales of the MFI, indicating the diagnosis of fatigue syndrome. GF: General fatigue. MF: Mental fatigue. MFI: Multidimensional Fatigue Inventory. PhF: Physical fatigue. RA: Reduced activity. RM: Reduced motivation.

Young women had a significantly higher percentage of psychometric challenges and fatigue with scores ≥12 on the RA, PhF, and MF scales compared to young men (p=0.008, 0.006, and 0.0002, respectively) (Table 2 and Figure 1). Additionally, young women had significantly higher odds (OR) of RA (OR 2.4, p<0.0001), PhF (OR 2.5, p<0.0001), and MF (OR 3.4, p<0.0001) than young men (Table 3).

**Table 2:** MFI-scales (score ≥12) among young, middle-aged, and elderly women compared to men

MFI scales	Young women (n=223)	Young men (n=224)	p-value (X^2^ test)
General fatigue (GF) ≥12	49.8% (111/223)	47.8% (107/224)	p=0.8
Reduced activity (RA) ≥12	60.09% (134/223)	38.8% (87/224)	p=0.008*
Reduced motivation (RM) ≥12	55.2% (123/223)	42.9% (96/224)	p=0.1
Physical fatigue (PhF) ≥12	61.4% (137/223)	38.8% (87/224)	p=0.006*
Mental fatigue (MF) ≥12	62.3% (139/223)	33.04% (74/224)	p=0.0002*
**MFI scales**	**Middle-aged women (n=348)**	**Middle-aged men (n=347)**	
General fatigue (GF) ≥12	58.6% (204/348)	40.6% (141/347)	p=0.005*
Reduced activity (RA) ≥12	52.01% (181/348)	46.4% (161/347)	p=0.38
Reduced motivation (RM) ≥12	59.5% (207/348)	41.2% (143/347)	p=0.005*
Physical fatigue (PhF) ≥12	59.5% (207/348)	41.2% (143/347)	p=0.005*
Mental fatigue (MF) ≥12	56.8% (201/348)	42.1% (146/347)	p=0.01*
**MFI scales**	**Elderly women (n=230)**	**Elderly men (n=220)**	
General fatigue (GF) ≥12	66.5% (153/230)	35.5% (78/220)	p=0.0001*
Reduced activity (RA) ≥12	51.7% (119/230)	46.4% (102/220)	p=0.5
Reduced motivation (RM) ≥12	67.4% (155/230)	37.3% (82/220)	p=0.0003*
Physical fatigue (PhF) ≥12	65.7% (151/230)	36.8% (81/220)	p=0.0004*
Mental fatigue (MF) ≥12	55.2% (127/230)	43.6% (96/220)	p=0.1

* : Significant difference. Fatigue syndrome was diagnosed at a score ≥12. Data is presented as numbers and percentages (%). Elderly: >60-75 years old. MFI: Multidimensional Fatigue Inventory. Middle age: >44-60 years old. X^2^: The chi-square test was used for the analysis of qualitative data. Young age: >18-44 years old.

**Table 3:** MFI-scales (score ≥12) among young women compared to young men

MFI scales	Young women (n=223)	Young men (n=224)	p-value (OR 95% CI)
**General fatigue (GF) ≥12** - Positive - Negative	111 112	107 117	p=0.7 (1.1; 0.75-1.6)
**Reduced activity (RA) ≥12** - Positive - Negative	134 89	87 137	p<0.0001* (2.4; 1.6-3.5)
**Reduced motivation (RM) ≥12** - Positive - Negative	123 100	96 128	p=0.009* (1.6; 1.13-2.4)
**Physical fatigue (PhF) ≥12** - Positive - Negative	137 86	87 137	p<0.0001* (2.5; 1.7-3.7)
**Mental fatigue (MF) ≥12** - Positive - Negative	139 84	74 150	p<0.0001* (3.4; 2.3-4.9)

* : Significant difference. Fatigue syndrome was diagnosed at a score ≥12. CI: Confidence Interval. MFI: Multidimensional Fatigue Inventory. The odds ratio was calculated using MedCalc 20.106. OR: Odds ratio. Young age: >18-44 years old.

**Figure 2:**
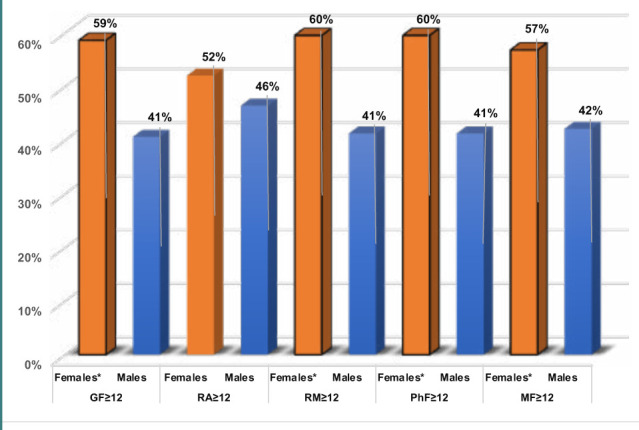
MFI-scales (score ≥12) among middle-aged women compared to middle-aged men *: Significantly different percentages between women and men with scores ≥12 on the GF, RM, PhF and MF scales of the MFI, indicating the diagnosis of fatigue syndrome. GF: General fatigue. MF: Mental fatigue. MFI: Multidimensional Fatigue Inventory. PhF: Physical fatigue. RA: Reduced activity. RM: Reduced motivation.

Furthermore, the percentage of middle-aged women who had scores ≥12 on the GF, RM, PhF, and MF scales was significantly higher (p=0.005, 0.005, 0.005, and 0.01, respectively) than middle-aged men (Table 2 and Figure 2). Additionally, middle-aged women had significantly higher odds of GF (OR 2.1; p<0.0001), RM (OR 2.1, p<0.0001), PhF (OR 2.1, p<0.0001), and MF (OR 1.9, p<0.0001) than did middle-aged men (Table 4).

**Table 4:** MFI-scales (score ≥12) among middle-aged women compared to middle-aged men

MFI scales	Middle -aged women (n=348)	Middle -aged men (n=347)	p-value (OR 95% CI)
**General fatigue (GF) ≥12** - Positive - Negative	204 144	141 206	p<0.0001* (2.1; 1.5-2.8)
**Reduced activity (RA) ≥12** - Positive - Negative	181 167	161 186	p=0.1 (2.4; 1.6-3.5)
**Reduced motivation (RM) ≥12** - Positive - Negative	207 141	143 204	p<0.0001* (2.1; 1.5-2.8)
**Physical fatigue (PhF) ≥12** - Positive - Negative	207 141	143 204	p<0.0001* (2.1; 1.5-2.8)
**Mental fatigue (MF) ≥12** - Positive - Negative	201 147	146 201	p<0.0001* (1.9; 1.4-2.5)

* : Significant difference. Fatigue syndrome was diagnosed at a score ≥12. CI: Confidence interval. MFI: Multidimensional Fatigue Inventory. Middle age: >44-60 years old. The odds ratio was calculated using MedCalc 20.106. OR: Odds ratio.

**Figure 3:**
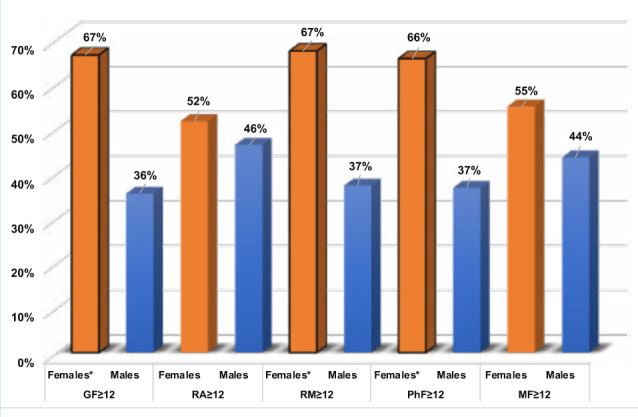
MFI-scales (score ≥12) among elderly women compared to men *: Significantly different percentages between women and men with scores ≥12 on the GF, RM and PhF scales of the MFI, indicating the diagnosis of fatigue syndrome. GF: General fatigue. MF: Mental fatigue. MFI: Multidimensional Fatigue Inventory. PhF: Physical fatigue. RA: Reduced activity. RM: Reduced motivation.

In the elderly age group, the percentage of elderly women who had a score ≥12 on the GF, RM, and PhF scales was significantly higher (p=0.0001, 0.0003, and 0.0004, respectively) than in older men (Table 2 and Figure 3). Additionally, elderly women had significantly higher odds of GF (OR 3.6, p<0.0001), RM (OR 3.5, p<0.0001), and PhF (OR 3.5, p<0.0001) than did older men (Table 5).

**Table 5:** MFI-scales (score ≥12) among elderly women compared to men

MFI-20 scales	Elderly women (n=230)	Elderly men (n=220)	p-value (OR 95% CI)
**General fatigue (GF) ≥12** - Positive - Negative	153 77	78 142	p<0.0001* (3.6; 2.5-5.3)
**Reduced activity (RA) ≥12** - Positive - Negative	119 111	102 118	p=0.3 (1.2; 0.86-1.8)
**Reduced motivation (RM) ≥12** - Positive - Negative	155 75	82 138	p<0.0001* (3.5; 2.4-5.1)
**Physical fatigue (PhF) ≥12** - Positive - Negative	155 75	81 139	p<0.0001* (3.5; 2.4-5.2)
**Mental fatigue (MF) ≥12** - Positive - Negative	127 103	96 124	p=0.01* (1.6; 1.1-2.3)

* : Significant difference. Fatigue syndrome was diagnosed at a score ≥12. CI: Confidence interval. Elderly: >60-75 years old. MFI: Multidimensional Fatigue Inventory. The odds ratio was calculated using MedCalc 20.106. OR: Odds ratio.

## Discussion

The Covid-19 pandemic has emerged as one of the most defining events of the 21^st^ century, causing widespread fear and uncertainty [25]. While many previous studies have focused on assessing participants' knowledge of preventive measures against COVID-19, there has been limited research on the behavioral changes of individuals during the pandemic. Therefore, this study aimed to identify the population at risk of experiencing psychometric challenges and fatigue during the COVID-19 pandemic in Kazakhstan.

We observed significant gender-related differences in employment status, with a higher percentage of working men compared to working women and a higher proportion of retired women compared to men. These disparities can be attributed to cultural and traditional beliefs that often assign women the role of family caretakers at home, while men are expected to be the primary breadwinners and financial supporters of the family [26].

The significantly higher percentage of previously COVID-19-infected men compared to women (p=0.02) can be explained by the higher percentage of working men compared to women in this study and the working environment. The risk of COVID-19 infection increased in poorly ventilated working environments with high population density and contact with the public for an extended period without personal protective equipment [27].

Our study revealed that women across different age groups exhibited significantly higher odds of experiencing various aspects of fatigue syndrome than men. Young women had significantly higher odds of RA, PhF, and MF than young men, and middle-aged women had higher odds of GF, RM, PhF, and MF than middle-aged men. Additionally, elderly women had significantly higher odds of GF, RM, and PhF than elderly men. This can be explained by the cultural and traditional beliefs that support women as caretakers of the family at home [26], with subsequent exposure to conflicting COVID-19 opinions and news from the media, including television, radio, and social networks [10]. Sadati *et al*. [25] found that the COVID-19 pandemic identified a risky society and vulnerable individuals exposed to more harm and suffering during the COVID-19 pandemic than others, including women, children, the elderly, individuals with disabilities or low income. Marshall *et al*. [28] also reported that pregnant women, families with children, elderly individuals, those with disabilities, and low-income individuals were socially, physically, and economically vulnerable during the COVID-19 pandemic. Furthermore, they found that vulnerable populations were less likely to take self-protective measures during and/or after the pandemic based on their knowledge and income, and they were at a higher risk of poor physical and psychological outcomes after the pandemic. Dorfan& Woody [8] reported several behavioral changes during the SARS outbreak, including avoidance, anxiety, the urge to wash, and increased wipe consumption. Elhadi *et al*. [9] studied self-preventive measures and behavioral changes during COVID-19 among college students and found that 71.8% of the participants were aware of COVID-19, and >92.7% of students (medical and non-medical) took preventive measures. Approximately 97% of the participants reported avoidance of crowds and shopping, which reflects high levels of caution against COVID-19. Furthermore, the authors found that the adoption of preventive measures and behavioral changes could be attributed to the knowledge acquired by college students and their exposure to high-risk environments [9].

Honarvar *et al*. [10] observed that most participants (69.1%) experienced negative effects on their routine activities due to the Covid-19 pandemic. Three-quarters of the participants stated that the Covid-19 pandemic negatively affected their routine lives. Participants preferred receiving COVID-19 news from national television/radio, social networks, and satellite channels [10]. The authors also explained the pandemic's negative effects on the participants' routines, linking these effects to their level of knowledge (i.e., 50% of the study population considered COVID-19 a deadly disease while fewer than 50% considered themselves at risk of contracting COVID-19).

This was the first cross-sectional cohort study conducted in the Republic of Kazakhstan to detect populations vulnerable to psychometric challenges and fatigue during the COVID-19 pandemic. We identified that women were more vulnerable, with significantly higher odds of different aspects of fatigue syndrome than men. Young women had significantly higher odds of RA, PhF, and MF than young men, and middle-aged women had higher odds of GF, RM, PhF, and MF than middle-aged men. Additionally, elderly women had significantly higher odds of GF, RM, and PhF than elderly men.

The impact of human behavior on the occurrence of health crises and pandemics is well-established [29]. The consequences of these behaviors have taken a toll on our environment, necessitating a fresh approach involving new dialogues about behavior and biology. Food and housing behaviors may play a crucial role in promoting the COVID-19 virus mutation and spreading. This study suggests an international program with an interdisciplinary management approach that includes sociologists, philosophers, epidemiologists, virologists, and public health experts to educate and motivate populations to change their behavior toward the environment. Furthermore, we advocate for substantial investments in risky societies to avoid the effects of conflicting opinions on vulnerable individuals. The media can increase the public’s awareness of maintaining physical and mental health and avoiding deterioration of emotional and behavioral well-being [30, 31].

Some of the limitations of this study include the limited availability of research on individual behavior changes during the COVID-19 pandemic and challenges related to participant refusal or communication difficulties.

## CONCLUSION

This study revealed that women exhibited greater vulnerability, with significantly higher odds of experiencing various aspects of fatigue syndrome than men during the COVID-19 pandemic in the Republic of Kazakhstan. This underscores the importance of investigating individual behavioral changes during pandemics, which can help identify vulnerable individuals and add valuable evidence for developing protocols and guidelines in pandemic and outbreak situations.
